# Evaluation of Three Adolescent Sexual Health Programs in Ha Noi and Khanh Hoa Province, Vietnam

**DOI:** 10.1155/2012/986978

**Published:** 2012-05-17

**Authors:** Van Pham, Hoang Nguyen, Le Huu Tho, Truong Tan Minh, Porntip Lerdboon, Rosemary Riel, Mackenzie S. Green, Linda M. Kaljee

**Affiliations:** ^1^Institute of Social and Medical Studies, Ha Noi, Vietnam; ^2^Tay Ho Clinics, Ha Noi, Vietnam; ^3^Khanh Hoa Provincial Health Services, Nha Trang, Vietnam; ^4^The International Rescue Committee, Public Health Advocacy, Silver Spring, MD 20910, USA; ^5^School of Nursing, Office of Global Health, University of Maryland Baltimore, Baltimore, MD 21201, USA; ^6^FHI 360, Health Service Research, Durham, NC 27713, USA; ^7^Pediatric Prevention Research Center, The Carman and Ann Adams Department of Pediatrics, Wayne State University, Detroit, MI 43201, USA

## Abstract

With an increase in sexual activity among young adults in Vietnam and associated risks, there is a need for evidence-based sexual health interventions. This evaluation of three sexual health programs based on the Protection Motivation Theory (PMT) was conducted in 12 communes in Ha Noi, Nha Trang City, and Ninh Hoa District. Inclusion criteria included unmarried youth 15–20 years residing in selected communes. Communes were randomly allocated to an intervention, and participants were randomly selected within each commune. The intervention programs included Vietnamese Focus on Kids (VFOK), the gender-based program Exploring the World of Adolescents (EWA), and EWA plus parental and health provider education (EWA+). Programs were delivered over a ten-week period in the communities by locally trained facilitators. The gender-based EWA program with parental involvement (EWA+) compared to VFOK showed significantly greater increase in knowledge. EWA+ in comparison to VFOK also showed significant decrease at immediate postintervention for intention to have sex. Sustained changes are observed in all three interventions for self-efficacy condom use, self-efficacy abstinence, response efficacy for condoms, extrinsic rewards, and perceived vulnerability for HIV. These findings suggest that theory-based community programs contribute to sustained changes in knowledge and attitudes regarding sexual risk among Vietnamese adolescents.

## 1. Introduction

### 1.1. HIV and Sexual Health Issues in Vietnam

As home to 60% of the world's population, an increasingly generalized HIV epidemic, and rapid social and economic changes, Asia remains a significant source of concern in relation to the HIV pandemic [1]. In Vietnam, all 61 provinces and 3 urban centers (Ho Chi Minh, Ha Noi, and Da Nang) have reported HIV/AIDS cases. Between 2000 and 2010, HIV prevalence among 15 to 49 year olds increased from 0.27% to 0.44% with rates over 1 percent in Ho Chi Minh City and northern coastal regions [2, 3]. Young Vietnamese adults are disproportionately affected by the HIV epidemic with 63% of known cases among 20 to 29 year olds [4].

In Vietnam as well as other countries in Asia, epidemiological trends for HIV indicate continuing [5] or increasing heterosexual transmission [6, 7]. At this time, approximately 2.3 males are infected for each female; however, this ratio is decreasing as heterosexual transmission becomes more common. Throughout Asia, women are often at risk of contracting HIV from husbands and boyfriends engaged in higher-risk behaviors (e.g., drug use, transactional sex) [8, 9].

 In addition to HIV, adolescents and young adults in Vietnam experience other adverse sexual health outcomes including unwanted pregnancy [10] and sexually transmitted infections (STIs). A recent population-based study among married women (ages 18 to 49 years) in rural Vietnam indicates that 8.3% of women had hepatitis B, 4.3% chlamydia, and 0.7% gonorrhea [11]. It is estimated that adolescent women account for at least one-third of abortions in Vietnam [10]. Abortion is legal in Vietnam, and first trimester abortion is relatively safe when performed by midlevel health providers or doctors [12]. However, younger and unmarried women are more likely to wait past the first trimester to visit a medical facility out of fear of being stigmatized and/or poor knowledge regarding indicators of pregnancy. Also, because of stigmatization, actual data on STIs among young adults and pregnancies among unmarried women are difficult to obtain as those affected tend to use private rather than public health facilities where government reporting is required [13, 14].

### 1.2. Sociocultural and Economic Change in Vietnam

Vietnam has undergone multiple economic and social reforms in the past 30 years, most notably since 1986 and the establishment of a more liberalized market system (“Doi Moi”) [15]. The country has experienced a significant decrease in economic and political isolation, moving rapidly toward inclusion in the global economy and resulting in the emergence of a growing middle class [16]. The health and well-being of working age adults is critical for continued economic development in transitional societies such as Vietnam. Throughout the world, HIV/AIDS and other adverse reproductive health-related outcomes have a significant negative impact on demographic and economic development [17].

Recent changes in sexual norms, particularly for men, regarding engagement in casual and transactional sexual relations have been related to the political-economic effects of Doi Moi and governmental policies which have led to increases in personal autonomy, economic growth, leisure time, and private spaces for sex [18]. Qualitative research with Vietnamese women suggest that they perceive extramarital affairs as increasingly common and linked to greater prosperity [19]. Adolescents in Vietnam, like elsewhere in Asia, are developing within sociocultural contexts significantly different from previous generations. Adolescents and emerging adults have increasing access to international media and the internet, have more economic resources, and are more mobile than in the past. Changes are also evident in these young people's engagement in relationships and premarital sexual behaviors [20].

### 1.3. HIV and Sexual Health Education in Vietnam

Sexual health and HIV prevention resources for Vietnamese youth include community-based programs, for example, through Youth Union activities, written materials and internet sites, and school-based curricula. Access to these programs, however, is unsystematic and affected by factors such as engagement in state-sponsored organizations, literacy levels, and school status. In relation to school-based HIV and reproductive health education, programming is limited in terms of materials, time allocation, and teacher training. The coursework is provided in mixed-gender classes, and a majority of teachers are women, decreasing likelihood that boys and young men will feel comfortable asking questions and participating in discussions.

In Vietnam, parent-child communication about relationships, sexuality, and associated health risks is often avoided or limited to parents simply telling their adolescents and young adult children not to have sex. Vietnamese parents often feel embarrassed talking about sensitive issues but also hold to beliefs that information about sexuality, pregnancy, and contraception is not appropriate for adolescent and young adult unmarried children. In addition, academic aspirations may overshadow the need for youth to obtain reproductive information, as parents perceive that young adults should not be (and therefore are not) engaged in relationships until after completing their studies [21–23].

### 1.4. Gender and HIV Prevention and Reproductive Health

Gender, as socially constructed roles and responsibilities and attitudes regarding the social position and rights of men and women, has been recognized as contributing to risk and protective factors for HIV transmission and other poor sexual health outcomes in a wide range of sociocultural contexts [24–27]. On a global basis, gender roles and responsibilities and inequalities affect both men's and women's sexual health and access to health services and treatment. Gender constructs result in differential knowledge and awareness of sexual health issues, ideologies, expectations, and norms for sexual behaviors, different motivations for sexual activity and relationships, and differential socio-cultural and economic power to negotiate sex and safer sex [28, 29].

As Vietnam becomes less economically and socially isolated, multiple contradictions are revealed within day-to-day experiences as well as in expectations and roles and responsibilities for young men and women at the social, familial, and individual levels. While the Vietnamese government party emphasizes equality for men and women, policies as well as social structures and cultural attitudes and beliefs continue to (re)construct gender roles in which women are to be compliant and deferential to men. Virginity is highly valued for women, creating situations in which women have a decreased sense of need for knowledge and decreased perceptions of vulnerability to sexual risk [30]. Unequal power discourages open discussion about sexuality within relationships and can also lead to coercion for noncondom use. Constructs of masculinity encourage young Vietnamese men to be self-reliant and take risks. For men, premarital relationships are acceptable and encouraged through peer expectations. These expectations include sexual norms in relation to men's engagement in casual and/or transactional partnerships as a way to gain sexual experience. Studies indicate that both men and women subscribe to these norms regarding men's sexual behavior, thus placing both the men and their current/future partners at risk for HIV and other STIs [18, 31].

In this paper, we will present and discuss outcome evaluation data for three adolescent sexual health intervention programs guided by the Protection Motivation Theory (PMT): (1) Vietnamese Focus on Kids (VFOK) (standard of care control); (2) the gender-focused Exploring the World of Adolescents curricula (EWA) with adolescents only; (3) EWA with adolescents, their parents, and local healthcare providers (EWA+). The original hypotheses for the project predicted that the gender-focused EWA and EWA+ interventions would result in significant improvement compared to VFOK. In this paper, changes were evaluated between baseline and postintervention (3, 6, and 12 months) within each program, and VFOK was compared to EWA and EWA+. Predicted changes include (1) increased knowledge about HIV, STIs, and pregnancy and contraceptives; (2) Protection Motivation Theory (PMT) constructs for sexual risk and protective behaviors including increased perceptions of severity, vulnerability, self-efficacy for condom use and abstinence, and response efficacy of condom use and decreased perceptions of intrinsic and extrinsic rewards and response costs for condom use; (3) decreased intention to engage in sex in the next three months.

## 2. Methods

### 2.1. Theoretical Approach

**Figure 1:**
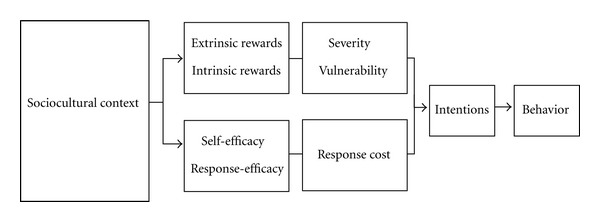
Protection Motivation Theory model.

Protection Motivation Theory (PMT) has been widely utilized for behavior-change program development and evaluation [32, 33] and is the basis for the US and Vietnamese Focus on Kids programs as well as the gender-focused EWA curricula [34]. Evaluation of the VFOK in the early 2000s indicated the appropriateness of the PMT for measuring attitudinal changes and predicting risk and protective intentions for Vietnamese youth [35]. PMT envisions environmental and personal factors through two appraisal pathways (threat and coping) which result in a maladaptive (risk) or adaptive (protective) response (Figure 1). The threat pathway is mediated by a balance between *intrinsic rewards* and *extrinsic rewards* accompanying the behavior, and *perceived severity *of and *perceived vulnerability* to the threat. *Intrinsic rewards* refer to internal perceived benefits of an action, while *extrinsic rewards* refer to peer and other social support for an action. *Severity *refers to an individual's perception of the degree to which an action will lead to significant negative physical, psychological, or social outcomes. *Vulnerability* refers to the perception of likelihood that one will personally experience negative outcomes.

The coping pathway is mediated by balancing perceived *self-efficacy* of a protective action, *response efficacy* of that action, and its *response cost*. *Self-efficacy* is defined as the perceived ability of an individual to perform a protective action, and *response efficacy* refers to the perceived effectiveness of those actions to decrease risks. *Response costs* are potential negative sequelae from engagement in protective behaviors. Socio-cultural context will affect which behaviors elicit positive and negative feelings, constrain or promote certain actions, and affect accessibility of information, goods, and services.

### 2.2. Interventions: Content and Implementation

Both VFOK and the EWA adolescent curricula include ten two-hour sessions, delivered once a week during the summer of 2006. The parent curriculum includes six two-hour sessions, which were simultaneously implemented with the youth program in EWA+ sites. Also at the EWA+ sites, a 2-day-training workshop was conducted for providers from the public commune health centers. The programs were delivered by trained community facilitators in local schools and community centers. The VFOK and the EWA adolescent curricula included sessions on basic knowledge about puberty, HIV, STIs, pregnancy, and contraceptives, and skills for decision making, communication, and condom use. EWA was composed of two separate curricula for young men and women and varied in activities, stories, and scenarios to target gender-specific sexual health issues as well as included activities focused on social constructs of gender. The EWA parent curricula focused on knowledge and parent-child communication. The EWA healthcare provider workshop focused on medical knowledge about sexual health issues, adolescent development, and communication. The sessions for each of the interventions were observed by Vietnamese and US research staff, and process evaluation forms were completed to record fidelity across interventionists and sites. These observations were discussed with facilitators at biweekly meeting to enhance delivery throughout the 10-week program. A full description of EWA program development and implementation is available elsewhere [36].

### 2.3. Research Sites

Program delivery and evaluation were conducted in four communes each in Ha Noi, Nha Trang City, and Ninh Hoa District in Khanh Hoa Province (total 12 communes). Sites represented different regions of the country and included both urban and rural areas. Communes were selected to represent a range of socio-economic conditions. Ha Noi is the capitol of Vietnam. Nha Trang is the provincial capital of Khanh Hoa Province in south-central Vietnam and is a popular beach resort with tourism its primary industry. Ninh Hoa is a rural district approximately 30 kilometers north of Nha Trang with an agricultural and aquacultural economic base.

### 2.4. Research Population Sampling and Recruitment

The evaluation utilized a clustered model with each of the 12 purposively selected communes randomized to one of the three intervention conditions (VFOK, EWA, and EWA+). Inclusion criteria included unmarried youth aged 15–20 years permanently residing in the research communes. Exclusion criteria included youth who would not be available to attend the ten-week programming or who had physical (e.g., were hearing impaired) or mental disabilities which precluded their ability to participate.

Among 2012 youth randomly selected based on household census data, 1020 (50.7%) were contacted. Primary reasons for not contacting youth were the household had moved or the youth was temporarily away at school or work. Among those contacted, 871 youth completed the baseline assessment (85.4%) with 317 youth allocated to VFOK, 281 youth to EWA, and 273 youth to EWA+. Sample size was calculated based on a procedure developed by Cohen [37] which estimates sample size as a function of effect size, alpha, power, and degrees of freedom. A sample size of 576 total participants would allow us to detect an effect size of  .20 (small effect). 

Community recruiters provided participants with information about the study and the specific intervention program to be implemented in that commune. Interested youth (and parents in the EWA+ communes) were scheduled to meet with project research staff to obtain additional information, be consented, and verify contact information.

The recruiters worked with the research staff during program implementation and the postintervention evaluation to remind participants of session times and data collection dates. For the youth program, mean overall attendance was 9 out of 10 sessions. There was no minimal requirement for attendance for inclusion in the analysis. There was a significant difference in median number of sessions attended by intervention group, with higher attendance for VFOK (9, IQR 6 to 10) and EWA (9, IQR 7 to 10) compared to EWA+ (8, IQR 6 to 10) (*P* = 0.002). Of the 273 youth participating in EWA+, 187 (68.5%) had parents attend at least one session and were included in the evaluation. Retention at 3, 6, and 12 months was 80%, 72%, and 57%, respectively.

### 2.5. Outcome Measures

The evaluation instrument was an expansion of previous instruments developed for the VFOK program. Additional modifications were made to the instrument based on qualitative interviews conducted prior to development of EWA. Instrument scales were piloted, and internal consistency (Cronbach's alpha) and test-retest reliability (Pearson's correlation coefficient) were checked. The instrument was also reviewed with groups of adolescents to ensure item clarity, intent, and readability. The following items and scales were used in the current analysis.

#### 2.5.1. Demographics

These include gender (male/female), age (continuous), school status (in/out of school), employment status (working/not working), religion (Buddhist, catholic, ancestor worship, and no religion), personal items (bicycle, motorbike, mobile phone, and computer), household items (telephone, DVD player, CD player, microwave, gas stove, refrigerator, washing machine, cable television, and computer), and personal monthly expenditures (continuous).

#### 2.5.2. Knowledge

Three scales were used with dichotomous responses (“true” and “false”) including 22 HIV/AIDS items, 11 STI items, and 10 pregnancy and contraceptive items.

#### 2.5.3. Protection Motivation Theory

Ten scales were developed including subscales for the constructs “severity,” “vulnerability,” and “self-efficacy.” Response items for the two self-efficacy scales were yes and no. For the other scales, response items were strongly agree, agree, neither agree nor disagree, disagree, strongly disagree.

Within the PMT threat appraisal pathway, the intrinsic rewards scale included 5 items (*α* = 0.85) (example: *my age would want to have sex to see how it feels*). The extrinsic rewards scale included 6 items (*α* = 0.68) (example: *my partner would love me more if I agree to have sex*). The severity for pregnancy subscale included 4 items (*α* = 0.86) (example: *if a girl is pregnant and unmarried, she must run away from her commune*). The severity for HIV/AIDS/STIs subscale included 7 items (*α* = 0.74) (example: *if I became infected with STI, I could have difficulty having children in the future*). The vulnerability for having sex subscale included 9 items (*α* = 0.67) (example: *movies will influence youth to have sex*). The vulnerability for HIV/AIDS/STIs included 8 items (*α* = 0.55) (example: *if I have unprotected sex I could contract HIV*). Despite modifications to the HIV/AIDS/STIs vulnerability scale, we were unable to increase the alpha above 0.55.

Within the PMT coping appraisal pathway, the condom use self-efficacy scale included 10 items (*α* = 0.88) (example: *I could put a condom on correctly*). The abstinence self-efficacy scale included 5 items (*α* = 0.87) (example: *I can wait until I am married before I have a sexual relationship*). The response efficacy scale included 3 items (*α* = 0.81) (example: *if you are going to have sex, condoms are an important way to prevent pregnancy*). The response cost scale included 5 items (*α* = 0.68) (example: *condoms take away the physical pleasure a boy has during sex*).

#### 2.5.4. Intentions and Behaviors

Respondents were asked their intentions to have sex in the next three months. Five response items ranged from very unlikely to very likely. Respondents were asked if they ever had vaginal sex (yes/no), if they had vaginal sex in the past 3 months (yes/no), number of lifetime partners (continuous), and frequency of condom use (always, more than half the time, half the time, rarely, or never).

### 2.6. Data Collection

Baseline data were collected in May and June 2006 with three postintervention followups at 3, 6, and 12 months. Data were collected at commune health centers and at youths' homes. To ensure greater confidentiality, data collectors read each item and response options, and respondents marked their answer on a separate copy of the survey. Overall 98.1% of respondents were judged by the data collectors as being average or above average readers. Respondents received a stipend (~$3.00) at each data collection point.

### 2.7. Data Management and Analysis

Data were double entered into SPSS data entry program (version 4.0) by trained staff. Variables were created for each PMT scale/subscale. The three knowledge scales were scored (correct/incorrect), and a total score was calculated for each scale. A socio-economic status scale was computed from principal component analysis (PCA) estimation on in-school status, employment, personal items, and household items. These estimated scores were ordered into quartiles.

Descriptive analysis of demographic data included frequencies, means, and medians. Knowledge and PMT constructs were created by summing items in each construct and then converting to standard scores ranging from 1 to 100. Standard scores conserve the characteristics of the original distribution and make analysis results more easily readable.

Analyses were conducted to both assess changes over time within each intervention and comparison of changes between VFOK and EWA, VFOK and EWA+. To check the equivalence of baseline characteristics between intervention groups, we used Wilcoxon rank-sum test for continuous variables and chi-square for categorical variables. To identify difference among intervention groups at each of evaluation times, we used repeated ANOVA tests.

Because communes were randomly assigned into intervention groups (clustered) and each participant had three followup evaluations, intervention effects in multivariate analysis were assessed using multilevel model. Multilevel model (MLM) (1) allows for assessment of main effect in the presence of confounders; (2) provides the capability to control for both cluster effect (community level) and highly correlated observations overtime on the same individual (individual level); (3) can handle incomplete data in repeated measures [38]. Each of the knowledge and PMT construct scales were put into the model as a dependent variable, and predictors included intervention groups (EWA, EWA+ versus VFOK); evaluation times (at months 3, 6, and 12 versus baseline); interaction between intervention groups and evaluation times; age in years; sex (male versus female); religious groups (any religion versus nonreligion); SES groups (richest (5th quintile) through poor (2nd quintile) versus poorest (1st quintile)) [39]. All *P* values reported were 2-sided, and <0.05 level was considered statistically significant.

### 2.8. Ethics

The project was approved by the institutional review board of the University of Maryland (Baltimore) School of Medicine and the ethical committee of Khanh Hoa Provincial Health Services. Participants under 18 years signed an assent form, and their parent/guardian signed a consent form. All other participants signed a consent form.

## 3. Results

### 3.1. Demographics

Participant median age was 17 years (IQR: 16–19), and 48.3% (421) of respondents were males. Seventy eight percent of respondents were in school (679/871) and 21.6% (188/871) were employed. The primary reported religions were Buddhism (44.2%), ancestor worship (19.6%), and catholicism (6.3%). Thirty percent of respondents reported no religious affiliation. Eighty eight percent of respondents lived with both parents. Mean monthly expenses were 150,000 VND (~US$10). Comparison across intervention groups reveals statistically significant differences for school status, employment, religion, and household items (see Table 1). 

### 3.2. Engagement in Relationships and Sexual Behaviors

At baseline, 40.4% (352/871) of respondents reported ever having a boy/girlfriend. Overall, 18.8% (79/421) male and 9.6% (43/450) female respondents reported ever engaging in sexual touching (*χ* ^2^ = 15.315, df1:  *P* < 0.001), and 6.4% (27/421) male respondents and 1.3% (6/451) female respondents reported ever engaging in vaginal sex (*χ* ^2^ = 15.450, df1:  *P* < 0.001). Nearly 8% (33/421) males compared to 1.5% (7/450) females reported intention to have sex in the next three months (*χ* ^2^ = 52.967, df3:  *P* < 0.001). Among sexually active respondents, 57.6% (19/33) reported using condoms half the time or less and 50% had 2 or more lifetime sexual partners.

### 3.3. Changes in Knowledge and PMT Construct Scores by Intervention Group

Mean change was analyzed for knowledge and PMT construct scores and intention to have sex between baseline and each follow-up period by intervention group. Effect was adjusted for age, gender, religion, and socio-economic status (based on accessible household items, monthly expenses, school, and employment status).

**Figure 2:**
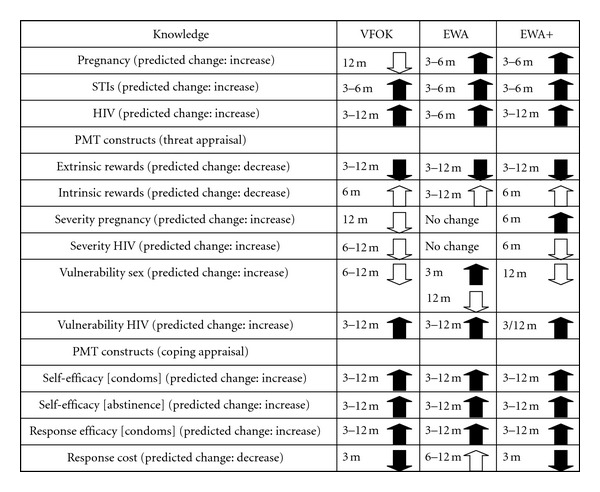
Statistically significant change (*P* < 0.05) for knowledge and PMT constructs by intervention (3, 6, and 12 months after intervention).

VFOK participants exhibited a sustained increase in knowledge for HIV/AIDS and up to 6 months for STIs. Among PMT threat appraisal constructs, there is a 12-month sustained significant decrease for extrinsic rewards and increase in perceived vulnerability to HIV. Among PMT coping constructs, there are 12-month sustained significant increases in self-efficacy for condom use and abstinence, and response efficacy of 12 months. There is also a significant decrease in perceived response costs at 3 months (Table 2 and Figure 2). 

Among the VFOK participants, there are other significant differences which are in the opposite direction from planned change including a decrease in knowledge about pregnancy and contraceptives, an increase in intrinsic rewards, and decreases in perceptions of pregnancy severity, HIV severity, and vulnerability to having sex.

For EWA participants, there are 6-month sustained significant increases in knowledge for pregnancy and contraceptives, STIs, and HIV/AIDS. There is a 12-month sustained decrease in extrinsic rewards and increase in perceived vulnerability for HIV/AIDS. Vulnerability for sex increases at 3 months, but then decreases at 12 months. There are sustained increases for self-efficacy condom use, self-efficacy abstinence, and response efficacy through 12-month followup. There is also unplanned change with intrinsic reward increasing through 12 months and response costs increasing at 6 and 12 months (Table 3 and Figure 2).

For the EWA+ program participants, there are 12-month sustained increases in knowledge for both pregnancy and contraceptives and HIV/AIDS and 6-month sustained increases in STI knowledge. There is a 12-month sustained decrease in extrinsic rewards. There are increases in perceived severity for pregnancy at 6 months and vulnerability to HIV/AIDS at 3 and 12 months. There are 12-month sustained increases for self-efficacy condom use, self-efficacy abstinence, and response efficacy. There is also a decrease in response cost at 3 months. Like VFOK and EWA, there are unanticipated changes including an increase in intrinsic rewards and decreases in perceived severity of HIV/AIDS and vulnerability for having sex (Table 4 and Figure 2). 

### 3.4. Intentions to Have Sex

**Figure 3:**
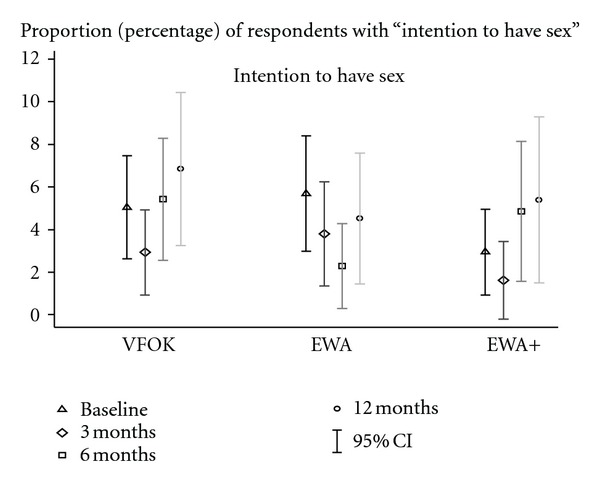
Percent of respondents with intention to have sex in the next 3 months by intervention (VFOK, EWA, and EWA+) and data collection point (3, 6, and 12 months).

The small number of youth who intended to have sex limited our evaluation of this variable. Comparing baseline to immediate postintervention (3 months) followup, all three interventions show decreases in intention to have sex. However, only EWA+ has a significant decrease after adjusting for age, gender, religion, and socio-economic status (*P* < .05). Both VFOK and EWA+ also show significant change after adjustment between baseline and 6 months after intervention; however, in both cases, there is an increase in intention to have sex (see Figure 3). 

### 3.5. Comparison of EWA and EWA+ to VFOK

The VFOK program served as the “standard of care” control. EWA+ participants compared to VFOK participants had a significantly greater increase in pregnancy and contraception knowledge at 3, 6, and 12 months and in STI knowledge at 12 months. There is no difference in HIV knowledge comparing EWA+ and VFOK; however, VFOK participants show significantly higher scores than EWA participants through 12-month followup. For the PMT constructs and intention to have sex, there are no significant differences comparing VFOK to either EWA or EWA+ (see Table 5). 

## 4. Discussion

Vietnam has experienced rapid social and political-economic changes over the past few decades. Within the context of these changes, adolescents and emerging adults have significantly greater access to knowledge and resources than their parents' generation, and sexual norms and the social constructs of gender continue to evolve within often conflicting contexts of “traditional” and “modern” values and beliefs [18–20, 40]. Our baseline data reveal low rates of engagement in sexual behaviors among our cohort of 15–20 year olds. Recent research suggests that sexual debut among Vietnamese youth occurs at 22.7 years for females and 21.3 for males [35]. Among males, data indicate rapidly increasing rates of engagement in nonmarital sexual behaviors from under 9% at 20 years to over 33% at 24 years. In addition, young men are engaging in casual and transactional sexual relations and inconsistent condom use [41, 42]. Furthermore, data on HIV prevalence as well as STIs and unwanted pregnancy among Vietnamese youth indicate a clear need for both early prevention programming before sexual debut as well as interventions for older adolescent and emergent adults.

We have presented the outcomes of a cluster-designed randomized evaluation for three HIV risk and sexual health interventions for Vietnamese adolescents. Changes within the PMT coping appraisal constructs include self-efficacy for both condom use and abstinence and response efficacy for condoms. These positive changes are sustained through 12-month postintervention followup. Among threat appraisal pathway constructs, across each intervention, there was a 12-month sustained decrease in extrinsic rewards. There were also sustained increases in perceived vulnerability to HIV. Other outcomes within the threat appraisal pathway constructs are less conclusive. Across all interventions, there is an increase from baseline to postintervention followups for “intrinsic rewards.” These data could reflect a need to reassess the scale items to better reflect cultural perceptions of “intrinsic rewards” and/or suggest that participation in a program in which sexual behaviors are openly discussed could empower youth to be more open about their sexual feelings. In both VFOK and EWA+, there are decreases in perceived severity of HIV/AIDS. Again, these changes could reflect a need for reassessing the scales and/or indicate at postintervention less HIV-associated stigma and therefore less severe perceived social consequences. Overall, these findings indicate more robust and sustained changes among constructs within the PMT coping appraisal pathway compared to the threat appraisal pathway. Such findings may indicate a need to strengthen programmatic content focused on rewards, severity, and vulnerability. Alternatively, these data may also reflect the coping appraisal constructs as more salient within Vietnamese culture—future cross-cultural data analysis may help determine patterns of construct relevancy outside of western contexts.

Outcomes data show decreased intention to have sex in the next three months for EWA+ participants at immediate postintervention (3 months); however, through 12 months participants across interventions show increases in intention to have sex. Without an assessment-only control group, this increase is difficult to interpret though it could reflect aging and maturity of the participants. With a control group, we would be able to compare if the increase among intervention participants was significantly less than control.

Comparison of VFOK and EWA indicates a 12-month sustained increase in HIV/AIDS knowledge among VFOK participants. This greater impact among VFOK participants could reflect more concentration on fact-based activities and less abstract concepts, for example, regarding gender roles and relationships, within the VFOK curriculum. Comparison of VFOK and EWA+ indicates a significantly greater increase in pregnancy and contraceptive knowledge among EWA+ participants at 3, 6, and 12 months and greater change in STI knowledge at 12 months. These data could indicate that parents participating in the EWA program became more communicative about sexual health knowledge. A lack of significant differences between EWA+ and VFOK in PMT constructs may reflect Vietnamese parents' greater comfort level sharing with their child new fact-based information compared to skill-based information (e.g., condom use). The EWA+ intervention included both a parent curriculum and a sexual health workshop for commune health center staff. Analysis of the parent evaluation data (reported elsewhere) indicates that parents improved significantly on knowledge about HIV, STIs, and pregnancy after intervention and level of comfort communicating with youth about sexual issues [43]. While it is not possible to discriminate if the parent and/or the health staff intervention may have contributed to the increases in knowledge among EWA+ participants, the data suggest that a multiple-level intervention including parents and/or broader community institutions has potential for the sexual health education of Vietnamese youth. A majority of Vietnamese youths live with both parents, and parents remain an untapped source for sexual health education. In addition, commune health workers have regular contact with residents and are engaged in intervention activities. Ensuring health workers have accurate information are comfortable communicating about sexual issues, and are essential components for improving sexual health of youth and the broader community.

At this time, there are limited numbers of evidence-based sexual health and HIV risk reduction programs for adolescents and emerging adults throughout low- and middle-income countries [44]. For Vietnamese youth, sexual health and HIV prevention resources remain limited in accessibility and scope. In order to facilitate future dissemination and implementation of efficacious programming, strides need to be made both in the adaptation and/or development of interventions and in the conduct of randomized-control evaluations of promising interventions. Overall, evaluation data indicate that each of these programs has promise; however, the EWA and EWA+ programs improve on the VFOK both in terms of sustainability and evidence of positive planned changes. The EWA adolescent curricula for males and females included gender-specific activities and issues which may have more effectively targeted the PMT constructs which can be affected by social contexts and constructs, for example, gender roles and relationships.

There is an unmet need for longer-term follow-up intervention assessments for adolescents in Vietnam and elsewhere in Asia. Extended follow-up assessment could provide data on whether or not the changes sustained through 12 months would be further sustained over time and delay sexual debut or increase engagement in safer sexual practices among young Vietnamese men and women. Further research is also needed on evidence-based program implementation and dissemination to increase youth and parent program participation within the contexts of school and work obligations, as well as cultural constructs and social norms which deny the need for sexual health knowledge prior to marriage.

## 5. Limitations

Due to the small percentage of participating youth reporting sexual activity during the 12-month evaluation, we were unable to analyze engagement in sexual behaviors, intention to use condoms, or condom use. Although we conducted the program during summer months to minimize conflicting school obligations, postintervention evaluations were conducted at times when youth would have been in school and older youth away from home at university. We experienced a significant drop in participation at 12 months which could have affected outcome data. We do not have specific information about differences between those youth who agreed to participate in the baseline evaluation and intervention and those who were unavailable or refused. In addition, the EWA+ intervention did not allow us to separately discern influences of health providers and parents.

## Figures and Tables

**Table 1:** Baseline demographic characteristics by intervention groups.

Variables	VFOK (*n* = 317)	EWA (*n* = 281)	EWA+ (*n* = 273)	Overall (*n* = 871)	*P* value^a^
Age in years median (IQR)	17 (16–19)	17 (16–19)	17 (16–19)	17 (16–19)	0.08
Male, *n* (%)	150 (47.3)	132 (47.0)	139 (50.9)	421 (48.3)	0.59
In school, *n* (%)	230 (72.6)	223 (79.4)	226 (82.9)	679 (78.0)	0.01
Employed, *n* (%)	85 (26.8)	56 (19.9)	47 (17.2)	188 (21.6)	0.01
Religion, *n* (%)					
Buddhist	144 (45.4)	116 (41.3)	125 (45.8)	385 (44.2)	0.01
Ancestor	73 (23.0)	60 (21.4)	38 (13.9)	171 (19.6)	
Catholic	23 (7.3)	10 (3.6)	22 (8.1)	55 (6.3)	
Nonreligion	77 (24.3)	95 (33.8)	88 (32.2)	260 (29.9)	
Living w/both parents, *n* (%)	283 (89.3)	247 (87.9)	240 (87.9)	770 (88.4)	0.83
Accessible household items, median (IQR)	5 (3–7)	5 (2.5–7)	6 (4–8)	5 (3–7)	0.01
Monthly expense in 1000 Vietnamese Dong, median (IQR)	150 (90–300)	155 (100–300)	150 (100–300)	150 (100–300)	0.28

^a^ Reported *P* values were from Wilcoxon rank-sum test for comparing continuous variables and chi-squared for categorical variables. *P* < 0.05 was considered to be statistically significant.

EWA: Exploring the World of Adolescents (youth intervention only); EWA+: Exploring the World of Adolescents (youth, parent, and healthcare provider interventions); IQR: Interquartile Range; VFOK: Vietnamese Focus on Kids.

**Table 2:** Standard mean scores (with standard deviation) (range 0–100) for knowledge and PMT constructions at baseline and followups for VFOK^a^.

	Baseline	3 months	6 months	12 months
*Knowledge*				
Pregnancy/contraceptives	51.45 (16.54)	52.84 (17.18)	52.58 (15.89)	47.54 (18.47)**
STIs	57.53 (18.55)	65.35 (17.06)***	62.14 (19.93)**	56.32 (20.90)
HIV/AIDS	56.76 (19.24)	71.06 (20.52)***	65.26 (23.66)***	65.54 (20.75)***

*PMT-threat appraisal*				
Extrinsic rewards	35.70 (18.58)	27.24 (17.38)***	28.08 (17.40)***	24.25 (14.68)***
Intrinsic rewards	38.83 (22.28)	39.43 (21.96)	47.80 (24.07)***	39.29 (20.18)
Perceived severity-pregnancy	31.90 (21.09)	31.16 (17.12)	32.38 (19.32)	27.04 (17.39)**
Perceived severity-HIV/AIDS	48.28 (17.45)	47.85 (15.42)	45.00 (17.48)***	45.34 (16.55)**
Perceived vulnerability-sex	45.19 (16.15)	44.50 (14.27)	42.91 (18.63)*	37.81 (14.69)***
Perceived vulnerability-HIV/AIDS	42.41 (16.21)	56.90 (17.79)***	48.27 (19.71)***	52.59 (18.94) ***

*PMT-coping appraisal*				
Self-efficacy condom use	50.35 (33.45)	76.63 (24.32)***	83.80 (21.66)***	86.63 (18.35)***
Self-efficacy abstinence	80.95 (31.36)	89.96 (23.96)***	90.25 (22.92)***	91.16 (23.04)***
Response efficacy	71.19 (23.34)	81.23 (18.89)***	76.79 (21.86)**	77.50**
Response cost	36.06 (17.32)	33.49 (19.17)*	38.28 (18.24)	35.76 (15.76)

^a^Significance calculated on adjusted mean change between baseline and each followup. Multilevel modeling (MLM) analysis adjusted for intervention group, evaluation times, interaction between intervention group and evaluation times, age, gender, religious group (any religion versus nonreligion), and socio-economic status.

*P < .05; **P < .01; ***P < .001.

PMT: Protection Motivation Theory; VFOK: Vietnamese Focus on Kids.

**Table 3:** Standard mean scores (with standard deviation) (range 0–100) for knowledge and PMT constructions at baseline and follow-ups for EWA^a^.

	Baseline	3 months	6 months	12 months
*Knowledge*				
Pregnancy/contractive	50.18 (15.38)	55.25 (18.16)***	54.45 (18.32)**	48.99 (20.36)
STIs	54.36 (19.16)	64.29 (17.83)***	60.72 (18.41)***	55.85 (19.40)
HIV/AIDS	54.94 (17.55)	63.61 (20.33)***	58.65 (21.34)*	60.00 (21.24)

*PMT-Threat Appraisal*				
Extrinsic rewards	33.72 (18.27)	29.05 (17.39)***	27.88 (17.16)***	25.52 (16.20)***
Intrinsic rewards	35.69 (20.90)	39.37 (20.93)**	46.95 (23.33)***	39.29 (18.71)**
Perceived severity-pregnancy	30.78 (17.15)	31.94 (16.14)	31.47 (16.88)	29.70 (16.85)
Perceived severity-HIV/AIDS	46.76 (16.52)	46.74 (14.19)	45.14 (16.03)	47.65 (15.71)
Perceived vulnerability-sex	42.78 (14.61)	45.06 (13.93)**	43.32 (17.07)	36.93 (13.69)***
Perceived vulnerability-HIV/AIDS	42.79 (15.31)	55.01 (15.10)***	47.81 (17.96)***	53.11 (18.35)***

*PMT-Coping Appraisal*				
Self-efficacy condom use	51.35 (34.43)	78.74 (23.66)***	83.38 (24.63)***	83.24 (23.00)***
Self-efficacy abstinence	82.57 (31.39)	88.82 (25.41)**	89.41 (25.65)**	91.41 (23.42)**
Response efficacy	70.58 (22.27)	78.43 (22.43)***	77.89 (21.62)***	76.09**
Response cost	35.39 (17.13)	35.04 (16.76)	39.48 (18.91)***	37.66 (14.38)**

^a^Significance calculated on adjusted mean change between baseline and each followup. Multilevel modeling (MLM) analysis adjusted for intervention group, evaluation times, interaction between intervention group and evaluation times, age, gender, religious group (any religion versus nonreligion), and socio-economic status. *P < .05; **P < .01; ***P < .001.

EWA: exploring the world of adolescents (youth program); PMT: protection motivation.

**Table 4:** Standard mean scores (with standard deviation) (range 0–100) for knowledge and PMT constructions at baseline and follow-ups for EWA+^a^.

	Baseline	3 months	6 months	12 months
*Knowledge*				
Pregnancy/contractive	50.35 (15.74)	56.82 (16.04)***	57.42 (16.67)***	53.19 (18.49)*
STIs	57.14 (17.95)	67.14 (15.41)***	64.63 (16.06)***	59.34 (21.67)
HIV/AIDS	57.31 (18.19)	70.08 (17.89)***	64.07 (20.92)***	62.92 (21.24)***

*PMT-threat appraisal*				
Extrinsic rewards	32.02 (16.07)	26.86 (17.42)***	28.22 (16.90)*	26.03 (14.37)**
Intrinsic rewards	38.55 (20.48)	40.51 (17.46)	50.15 (21.24)***	39.81 (17.73)
Perceived severity-pregnancy	29.46 (17.04)	31.02 (14.25)	32.51 (17.38)**	27.93 (16.62)
Perceived severity-HIV/AIDS	47.72 (17.21)	46.26 (13.84)	43.48 (14.47)**	45.81 (13.70)
Perceived vulnerability-sex	45.15 (14.30)	45.91 (12.53)	41.23 (15.40)	**36.55 (12.82)^a^**
Perceived vulnerability-HIV/AIDS	46.43 (16.82)	57.68 (15.96)***	48.60 (18.27)	53.93 (18.07)***

*PMT-coping appraisal*				
Self-efficacy condom use	59.71 (30.96)	80.27 (16.73)***	88.42 (17.49)***	88.92 (16.34)***
Self-efficacy abstinence	88.21 (26.90)	93.26 (19.33)*	92.85 (20.48)*	94.46 (16.71)*
Response efficacy	73.55 (19.04)	81.27 (21.12)***	80.35 (18.90)***	78.59*
Response cost	37.05 (17.24)	31.86 (16.77)***	37.40 (16.71)	34.69 (13.64)

^a^Significance calculated on adjusted mean change between baseline and each followup. Multilevel modeling (MLM) analysis adjusted for intervention group, evaluation times, interaction between intervention group and evaluation times, age, gender, religious group (any religion versus nonreligion), and socio-economic status. *P < .05; **P < .01; ***P < .001.

EWA+: Exploring the World of Adolescents (youth, parent, and healthcare provider program); PMT: Protection Motivation Theory.

**Table 5:** Comparison of FOK and EWA/EWA+ intervention effect on knowledge through 12-month followup^a^.

Constructs	EWA versus FOK (mean difference (SE))	EWA+ versus FOK (mean difference (SE))
*Pregnancy and contraceptives *		
Baseline	−1.33 (1.40)	−1.48 (1.42)
At 3 months	2.01 (1.50)	3.83 (1.61)*
At 6 months	1.66 (1.57)	4.53 (1.70)**
At 12 months	0.57 (1.74)	6.00 (1.90)**

*Sexually transmitted infection *		
Baseline	−3.46 (1.49)*	−0.76 (1.52)
At 3 months	−1.43 (1.60)	1.70 (1.72)
At 6 months	−1.17 (1.67)	2.79 (1.81)
At 12 months	−0.27 (1.84)	4.07 (2.01)*

*HIV/AIDS *		
Baseline	−1.94 (1.61)	−0.45 (1.64)
At 3 months	−7.57 (1.70)***	−0.20 (1.81)
At 6 months	−6.50 (1.76)***	0.09 (1.88)
At 12 months	−6.13 (1.89)**	−0.08 (2.04)

^a^ The effect was adjusted for age, gender, religious status, and social economic status. *P < .05; **P < .01; ***P < .001.

EWA: Exploring the World of Adolescents (youth intervention only); EWA+: Exploring the World of Adolescents (youth, parent, and healthcare provider interventions); VFOK,Vietnamese Focus on Kids.
